# Blind Testing: DNA Barcoding Sheds Light Upon the Identity of Plant Fragments as a Subsidy for Cave Conservation

**DOI:** 10.3389/fpls.2018.01052

**Published:** 2018-07-24

**Authors:** Aline J. Ramalho, Daniela C. Zappi, Gisele L. Nunes, Mauricio T. C. Watanabe, Santelmo Vasconcelos, Mariana C. Dias, Rodolfo Jaffé, Xavier Prous, Tereza C. Giannini, Guilherme Oliveira, Ana M. Giulietti

**Affiliations:** ^1^Instituto Tecnológico Vale, Belém, Brazil; ^2^Museu Paraense Emílio Goeldi, Coord. Botânica, Programa Capacitação Institucional, Belém, Brazil; ^3^Speleology, Vale S.A., Nova Lima, Brazil

**Keywords:** amazon, barcoding, canga, caverns, conservation, micromorphology, rhizothemes, troglobites

## Abstract

Plants living above and around caves represent an important, albeit poorly studied, resource within cave ecosystems. The presence of plant material (root-like structures or rhizothemes, saplings, seeds, and seedlings) correlates positively with the biodiversity of the cave dwelling animals as shown for iron-ore caves in Carajás, Pará, Brazil. Plant material collected in caves has proven to be difficult to identify by traditional botanical methods, thus this research aims to provide a qualitative insight into the taxonomy and morphology of rhizothemes and other plant fragments found in the caves. The identification process used a combination of different molecular markers (ITS2, *rbc*L, and *trn*H-*psb*A) followed by a comparison of the sequences obtained against publicly available databases. The rhizothemes were submitted to micromorphological analysis to ascertain their putative root or stem origin and to compare their anatomy with known patterns found in the plant families or genera recovered through molecular matches. All studied samples were Angiosperms, mostly belonging to subclass Rosideae, within four orders: Malpighiales (Euphorbiaceae, Hypericaceae), Sapindales (Anacardiaceae and Sapindaceae), Myrtales (Myrtaceae), Fabales (Fabaceae), and only two belonging to subclass Asteridae, order Gentianales (Apocynaceae). Some of the samples were matched to generic level, with ITS2 being the best marker to identify the fragments because it shows high degree of sequence variation even at specific level and result reliability. All rhizothemes turned out to be roots, and correspondence was found between the existing literature and the individual anatomical patterns for the families and genera retrieved. DNA barcode has proved to be a useful tool to identify plant fragments found in this challenging environment. However, the existence of well curated, authoritatively named collections with ample biological information has proven to be essential to achieve a reliable identification.

## Introduction

Human history and cave environments have been entwined for tens of thousands of years, generating a long-term interest that pervades paleontology, archeology, and biology, culminating in the development of speleology, a science totally dedicated to the study of caves (Ruchkys et al., 2015). Caves have fascinated visitors and enticed their curiosity regarding these systems (Marra, 2009) that can be formed by dissolution, erosion or tectonic movements, over different types of rock such as carbonatic, siliciclastic and ferriferous (Gunn, 2005; Piló and Auler, 2009; Culver and White, 2012). The high degree of specialization found in caves both from the biological and geological viewpoint, allied and fomented by society’s interest in the subject, have led to the creation of laws and regulations aiming to protect the world’s speleologic heritage. In Brazil, there is specific legislation regarding the protection and use of natural cavities (Federal Decree, 6640/2008) following a series of specific directions (Ganem, 2009). While it is only possible to suppress or modify caves if these are found to be of low, medium or even high relevance, maximum relevance caves are to be left untouched alongside with their area of influence. This has resulted in scientific research being dedicated to study aspects that influence the richness of cave fauna, including troglobites, as these organisms are used to establish the biological importance and the level of protection assigned to a given cave (Culver and Pipan, 2009; Trajano and Bichuette, 2010; Souza-Silva et al., 2015; Jaffé et al., 2016).

**FIGURE 1:**
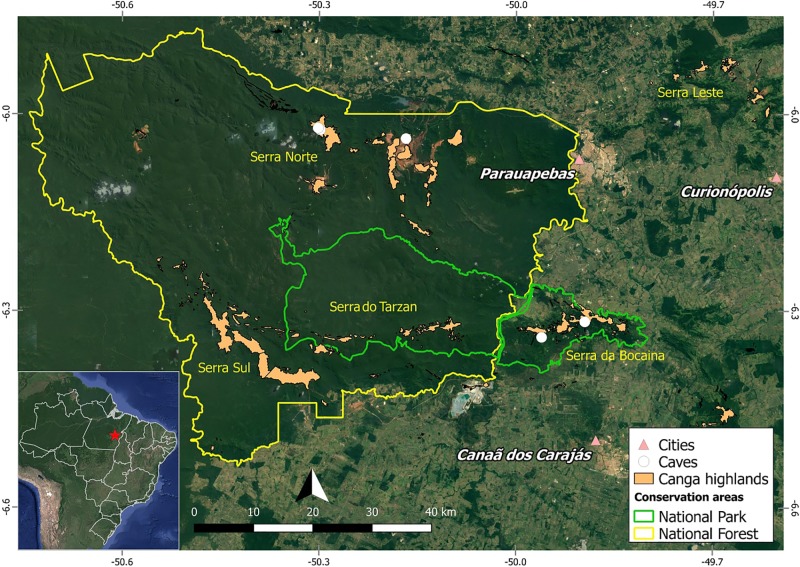
Map of Carajás showing localization of studied caves.

Occasional mention is made to seeds, roots, and plant debris in cave literature. Roots that grow around and above caves may reach different areas and depths, and are considered an important resource to maintain animal life (Howarth, 1973; Jasinska et al., 1996; Howarth et al., 2007; Silva et al., 2011). Stone et al. (2012) studied tree root communities and their interactions with troglobites within lava tubes. Brazilian researchers have compared the network of roots formed under water percolation inside caves with living sculptures reminiscent of speleothemes, and named them *rizotemas*, or rhizothemes (Ferreira, 2005). Considering iron-ore caves, Jaffé et al. (2016) studied animal species richness and concluded that there is a positive correlation between the presence of roots and faunistic diversity linked to a more abundant offer of plant resources. Plant roots are responsible for absorbing water and naturally display positive geotropism and negative phototropism. Roots found inside iron-ore caves and cavities belong to species that can tolerate the extreme conditions of the substrate, such as chemical composition, heat, flooding, and drought (Piló and Auler, 2009; Stone et al., 2012). Brazilian iron-ore outcrops are locally known as *canga* (Mota et al., 2015) and occur in Minas Gerais, Mato Grosso do Sul and in the region of the Serra dos Carajás, state of Pará (**Figure 1**). Vegetation found above these outcrops is an open scrub with occasional forest copses, while the cave openings tend to be at the edge of plateaus and surrounded by a denser Amazon Rainforest matrix that occurs on the slopes and lowlands (Viana et al., 2016). Caves found within this area were mapped and their animal diversity surveyed, and Jaffé et al. (2016) quantitatively studied roots and other plant fragments within the caves. Iron caves contain higher richness of troglomorphic species than caves of other lithologies (Silva et al., 2011) and, because they are located in areas of interest for mining companies, they are under severe economic pressure and are currently amongst the most threatened subterranean ecosystems (Auler and Piló, 2015).

The overall objective of this study is to provide a qualitative insight into the taxonomy and morphology of rhizothemes, bringing further information toward the study of the plant diversity and plant-animal interactions, both of value toward the conservation of this unique ecosystem and to inform the current procedures that are followed to ascertain the conservation of the local caves. The nature of the plant material available (sterile, fragmentary) is such that we attempt to use DNA barcoding with a combination of different molecular markers (ITS2, *rbc*L, and *trn*H-*psb*A) as a method to identify the specimens that cannot be named using traditional taxonomy (Miller, 2007; Hollingsworth et al., 2011).

## Materials and Methods

### Study Area

The caves studied are part of the Serra dos Carajás, located in the southeastern part of the state of Pará, Brazil, in the Floresta Nacional de Carajás (FLONA Carajás) and in the neighboring Serra da Bocaina, now part of the recently Parque Nacional dos Campos Ferruginosos (National Park of Ferruginous Fields), with seasonal climate with alternate dry (May to November) and rainy (December to April) seasons, and precipitation reaching 1,770 mm per year (Absaber, 1986; Mota et al., 2015). The *canga* outcrops in the region are estimated to house around two thousand caves (Piló et al., 2015). Five caves were surveyed for plant material collection, three at the Serra Norte of FLONA Carajás (Municipality of Parauapebas) in March 2016 (N1_0174, N1_0168, and N4E_0026), and two at the Serra da Bocaina (Municipality of Canaã dos Carajás) in June 2016 (SB_0049, SB_0212) (**Figure 1**). Coordinates are provided in **Table 1**.

**Table 1:** Specimens collected in five caves in two localities at the FLONA Carajás and Serra da Bocaina (PARNA Campos Ferruginosos) during 2016.

ID	Sample ID	Month	Locality	Cave	Latitude	Longitude	Location	Plant part
1	3718	March	FLONA Serra Norte	N1_0174	-6.024225	-50.298528	Semi-photic zone	rhizotheme
2	3719	March	FLONA Serra Norte	N1_0168	-6.021494	-50.301776	Dark zone	seedling
3	3720	March	FLONA Serra Norte	N1_0168	-6.021494	-50.301776	Dark zone	rhizotheme
4	3721	March	FLONA Serra Norte	N4E_0026	-6.037647	-50.167870	Semi-photic zone	rhizotheme
5	3722	March	FLONA Serra Norte	N4E_0026	-6.037647	-50.167870	Semi-photic zone	seed
6	3723	March	FLONA Serra Norte	N4E_0026	-6.037647	-50.167870	Dark zone	rhizotheme
7	3724	March	FLONA Serra Norte	N4E_0026	-6.037647	-50.167870	Dark zone	seedling
8	3725	June	Serra da Bocaina	SB_0049	-6.316629	-49.895138	Semi-photic zone	rhizotheme
9	3726	June	Serra da Bocaina	SB_0049	-6.316629	-49.895138	Semi-photic zone	rhizotheme
10	3727	June	Serra da Bocaina	SB_0049	-6.316629	-49.895138	Semi-photic zone	rhizotheme
11	3729	June	Serra da Bocaina	SB_0049	-6.316629	-49.895138	Semi-photic zone	rhizotheme
12	3730	June	Serra da Bocaina	SB_0049	-6.316629	-49.895138	Semi-photic zone	rhizotheme
13	3731	June	Serra da Bocaina	SB_0049	-6.316629	-49.895138	Semi-photic zone	rhizotheme
14	3732	June	Serra da Bocaina	SB_0049	-6.316629	-49.895138	Semi-photic zone	rhizotheme
15	3733	June	Serra da Bocaina	SB_0049	-6.316629	-49.895138	Semi-photic zone	rhizotheme
16	2770	June	Serra da Bocaina	SB_0212	-6.340371	-49.960872	Cave entrance	sapling and rhizotheme
17	2771	June	Serra da Bocaina	SB_0212	-6.340371	-49.960872	Cave entrance	sapling and rhizotheme


### Specimen Collection

A total of 17 plant fragments including 12 rhizothemes, two saplings (with leaves, two of them with rhizothemes), two seedlings (with seed still attached and leaves) and one seed (**Table 1**) were collected in 2016, as part of intensive fieldwork toward preparing the “Flora of the *cangas* of the Serra dos Carajás” (Viana et al., 2016). Concomitant botanical fieldwork also included surrounding areas. For the present research, the plant material was partly fixed in ethanol 70% for micromorphology analysis (rhizothemes only), and the remaining was stored in a NaCl-saturated solution of 2% CTAB (Rogstad, 1992) for molecular analyses. A single specimen, sample ID16 (**Table 1**), a rhizotheme with aerial parts (stem and leaves), had a voucher prepared (*Giulietti 2576*) and deposited at the herbarium of the Museu Paraense Emílio Goeldi (MG).

During the above mentioned floristic project, concomitant DNA collections were made for approximately two thousand voucher specimens deposited at the MG herbarium. The sequenced regions include ITS2, *rbc*L, and *trn*H-*psb*A, all recognized among the most promising markers for plant DNA barcoding. Barcodes of plants collected in the Serra dos Carajás region (Pará-Brazil) were preferably chosen as reference for this work. All sequences used in the work were deposited in public databases (BOLD and GenBank). The record numbers for BOLD are: ITVRT001-17 – ITVRT044-17; while GenBank accession numbers are: ITS2 (MG217353 – MG217392), *rbc*L (MG407424 – MG407458), and *trn*H-*psb*A (MG220397 – MG220413).

### DNA Extraction, Amplification and Sequencing

The total genomic DNA was extracted from plant fragments according to Weising et al. (2005) and stored at –20°C. Chloroplast genes (*trn*H-*psb*A and *rbc*L) and nuclear ribosomal intergenic spacer (ITS2) were amplified using previously published universal forward and reverse primers (**Table 2**). PCR amplifications were carried out with 2 μL of total DNA (∼10–100 ng), 1×*Taq* buffer with KCl, 3 μmol/mL MgCl_2_, 0.2 μmol/mL of dNTPs, 0.1 nmol/mL of each forward and reverse primer (**Table 2**) and 1 U of *Taq* DNA polymerase in a 25 μL aqueous solution. Amplification was carried out by thermal cycling with the following steps: denaturation at 94°C for 3 min, 35 cycles of 94°C cycles for 1 min, 46–54°C for 1 min and 72°C for 1 min with a final hold at 72°C for 7 min (modified from Shaw et al., 2007). DNA integrity was determined by electrophoresis in agarose gel containing SYBR Safe (Thermo Fisher Scientific, Carlsbad, CA, United States*).* Sequencing was carried out on an automatic sequencer ABI 3730 DNA Analyzer (Thermo Fisher Scientific) using the BigDye Terminator v3.1 Sequencing Standard Kit (Applied Biosystems), following the manufacturer’s instructions. Both strands of DNA were sequenced, and repeated if necessary.

**Table 2:** Primers used for DNA barcode production.

Region amplified	Primer	Sequence 5′-3′	Reference
*rbc*L	*rbc*LbF	AGACCTWTTTGAAGAAGGTTCWGT	Dong et al. (2014)
	*rbc*LbR	TCGGTYAGAGCRGGCATRTGCCA	
ITS2	ITS-S2F	ATGCGATACTTGGTGTGAAT	Chen et al. (2010)
	ITS3R	GACGCTTCTCCAGACTACAAT	
*trn*H-*psb*A	*trn*H	CGCGCATGGTGGATTCACAATCC	Tate and Simpson (2009) Sang et al. (1997)
	*psb*A	GTTATGCATGAACGTAATGCTC


Quality control and assembly of partial sequences generated by Sanger sequencing of ITS2, *rbc*L, and *trn*H-*psb*A markers was performed by Geneious R10 software (Biomatters, Auckland). The electropherograms were analyzed and trimmed using the *modified-Mott* algorithm with a 00.1 error probability limit. High quality sequences (phred > 20) were used for produce the consensus sequence (forward and reverse). The consensus sequences were generated individually for each specimens analyzed considering the particular marker.

A BLASTn search against NCBI database was performed in order to assign an identification to the samples (Altschul et al., 1990). Barcodes of plants collected in the Serra dos Carajás region (Pará-Brazil) were preferably chosen as reference for this work. When not possible to match the sample with local material, a wider search within GenBank was performed (**Supplementary Table S1** and **Table 3**). The complete correspondence between BOLD and GenBank numbers for our samples is in **Supplementary Table S2**).

**Table 3:** Identity of samples using three molecular markers (determinations that were matched against specimens collected locally by the Flora of Carajás project are highlighted in gray, matches against records from other locations, already available in the GenBank, are not highlighted).

			Marker
	**Sample ID**	**ITS2**	**rbcL**	**trnH**	
1	ITV3718	Fabaceae *Parkia* 83%(100)	Fabaceae *Camptosema* 97%(100)	Fabaceae *Parkia* 98%(52)	Root
2	ITV3719	–	Fabaceae *Dioclea* 97%(99)	Fabaceae *Aeschynomene* 91%(43)	Seed
3	ITV3720	Euphorbiaceae *Aparisthmium* 97%(100)	Euphorbiaceae *Aparisthmium* 100%(100)	–	Root
4	ITV3721	Fabaceae *Dioclea* 88%(100)	Fabaceae *Dioclea* 97%(100)	Fabaceae *Abrus* 94%(97)	Root
5	ITV3722	Myrtaceae *Myrcia* 99%(100)	–	–	Seed
6	ITV3723	Fabaceae *Dioclea* 88%(100)	Fabaceae *Dioclea* 97%(99)	Fabaceae *Abrus* 95%(97)	Root
7	ITV3724	Myrtaceae *Myrcia* 100%(99)	Myrtaceae *Myrcia* 100%(95)	Myrtaceae *Myrcia* 100%(100)	Seedling
8	ITV3725	Anacardiaceae *Tapirira* 99%(99)	Anacardiaceae *Tapirira* 99%(100)	–	Root
9	ITV3726	Anacardiaceae *Tapirira* 99%(97)	Anacardiaceae *Tapirira* 100%(100)	Anacardiaceae *Tapirira* 98%(99)	Root
10	ITV3727	Anacardiaceae *Tapirira* 100%(100)	–	–	Root
11	ITV3729	Anacardiaceae *Tapirira* 99%(100)	Anacardiaceae *Tapirira* 100%(100)	Anacardiaceae *Tapirira* 98% (100)	Root
12	ITV3730	Apocynaceae *Forsteronia* 100%(99)	Apocynaceae *Secondatia* 99%(99)	Apocynaceae *Odontadenia* 94%(89)	Root
13	ITV3731	Anacardiaceae *Tapirira* 100%(100)	Anacardiaceae *Tapirira* 100%(100)	Anacardiaceae *Tapirira* 98%(100)	Root
14	ITV3732	Sapindaceae *Serjania* 99%(66)	Sapindaceae *Serjania* 100%(100)	Sapindaceae *Serjania* 98%(94)	Root
15	ITV3733	Apocynaceae *Forsteronia* 100%(98)	Apocynaceae *Secondatia* 99%(100)	Apocynaceae *Odontadenia* 94%(89)	Root
16	ITV2770	Hypericaceae *Vismia* 100%(100)	–	–	Sapling and root
17	ITV2771	Hypericaceae *Vismia* 100%(100)	–	Hypericaceae *Vismia* 99%(84)	Sapling and root


– denotes the sequence was not amplified; values = % identity (% query coverage per subject).

### Rhizotheme Anatomy

Rhizotheme samples were transversally sectioned by hand at the apex and base of the structures and semi-permanent slides mounted with glycerinated gelatin. Images were obtained using AxioCam MRc Rev.5 FireWire digital camera adapted to a Zeiss 426126. Nomenclature used for the descriptions followed Metcalfe and Chalk (1950) and Fahn (1979).

## Results

### Molecular-Barcode

A total of 41 sequences were obtained for the 17 samples analyzed, including 16 for ITS2, 13 for *rbc*L and 12 for *trn*H-*psb*A. Only the first identity retrieved was included in **Table 3**, where the top match for each available marker is shown using only family and generic level. Further matches and scores can be seen in the supporting material (**Supplementary Table S1**).

### Sequence Analyses

BLASTN indicates the likely identification of the plant. To further confirm we conducted phylogenetic analysis of the sequences. In **Table 3** and **Supplementary Table S1** it can be seen that the different markers has distinct power for resolving the identification of the plant fragments.

The results indicate that the plant fragments studied were rhizothemes belonging to the families Anacardiaceae, Apocynaceae, Euphorbiaceae, Fabaceae, and Sapindaceae, while the seed and seedling were matched with Myrtaceae. Hypericaceae was represented by saplings attached to rhizothemes. At family level there was complete agreement between the local samples and the ones obtained from GenBank.

All samples were named to family level (seven families), and genera were suggested for all 17 of them, however, different generic matches were indicated depending on the samples available for different markers. Only samples with total agreement between markers were named to generic level: *Vismia* (Hypericaceae, samples 16 and 17), *Myrcia* (Myrtaceae, samples 5 and 7) and *Tapirira* (Anacardiaceae, samples 8–11 and 13). The highest number of different genera were suggested for the two Apocynaceae samples (samples 12 and 15 – *Forsteronia/Secondatia/Odontadenia*).

Cave N1_0174 had a single collection, the rhizotheme retrieved as Fabaceae (*Parkia/Camptosema*), while a seedling of Fabaceae (*Dioclea/Aeschynomene*) and a Euphorbiaceae (*Aparisthmium*) rhizotheme were collected in cave N1_0168. Cave N4E_0026 had one seed and one seedling of Myrtaceae (*Myrcia*) and two rhizothemes of Fabaceae (*Dioclea*/*Abrus*). All rhizothemes from one of the Serra da Bocaina caves (SB_0212) were retrieved by all markers as *Vismia* (Hypericaceae), while cave SB_0049 in the same locality yielded eight rhizothemes, five of *Tapirira* (Anacardiaceae), two of Apocynaceae (*Forsteronia/Secondatia/Odontadenia*) and one *Serjania* (Sapindaceae).

### Morphology

**FIGURE 2:**
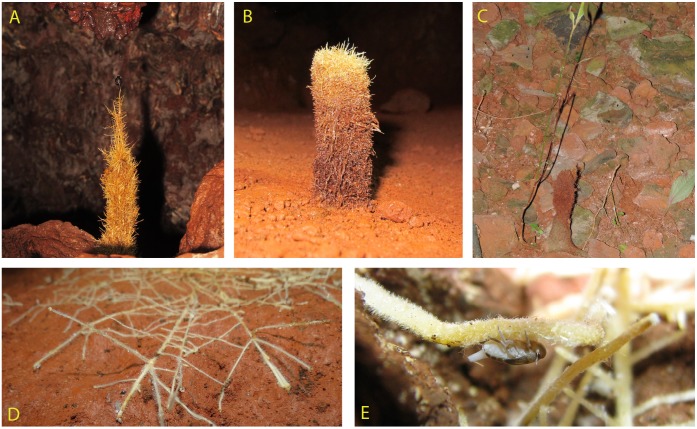
Diversity of roots and rhizothemes found in Carajás caves. **(A,B)** Candle-shaped, vertical rhizothemes. **(C)** Sample 16 showing rhizotheme attached to stem and leaf, collected at cave entrance. **(D)** Root network. **(E)** Detail showing insect-root interaction (photos X. Prous).

Rhizothemes with different macromorphology were studied in this work and are illustrated alongside roots in **Figure 2**. Some of them formed horizontal networks on the cave floor and walls (**Figure 2A**), while the more outstanding were vertical and branched and tended to grow upright under water percolation from the cave ceiling (**Figures 2A–C**). Roots in close up were hairy (**Figures 2D,E**). All rhizotheme sections examined are brownish in color, probably due to the presence of phenolic compounds, and the sections grouped by their identification are described as follows:

### Sample 4 – (Fabaceae subf. Faboideae)

**FIGURE 3:**
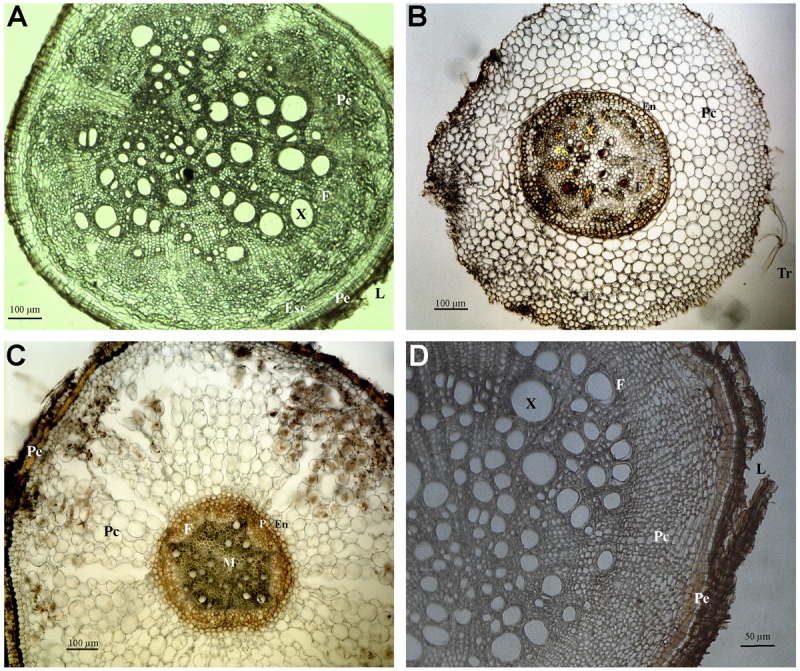
Photomicrographs of transverse sections of Fabaceae **(A,B)**, Apocynaceae **(C)** and Hypericaceae **(D)** roots. **(A)** Sample 4, secondary growth. **(B)** Sample 6, primary growth. **(C)** Sample 12, primary growth. **(D)** Sample 16, secondary growth. Abbreviations: Tr, root hair; Ep, epiderm; Ex, exoderm; Pe, periderm; Pc, cortical parenchyma; En, endoderm; P, pericycle; M, medulla; X, xylem; F, phloem; Es, secretory structure; Esc, schlerenchyma; L, lenticel.

Cylindric root displaying secondary growth (**Figure 3A**): periderm with lenticels and central cylinder with xylem, rays, and phloem. The cortex displays a sheath or sclerenchymal ring with ramiform punctuations, parenchymal cells intercalated with sclerenchymal fibers. The xylem cells are rounded, with wide lumen.

### Sample 6 – (Fabaceae subf. Faboideae)

Cylindric root displaying primary growth (**Figure 3B**): epiderm with root hairs, cortex with several layers limited interally by the pericycle, endoderm, and vascular cylinder. The peripheral area of the cortex is formed by parenchymal cells that increase in diameter closer to the vascular cylinder. The endoderm cells are hexagonal and compact, and the Caspary striations are U-shaped, opening toward the vascular cylinder. The pericycle has a layer of thin walled cells. Phloem is protected by fibrous shields with cellulose, lignine or suberine deposits. The root is tetrarch, and there are secretory cells in the parenchymal pith.

### Samples 8–11 and 13 – *Tapirira* (Anacardiaceae)

**FIGURE 4:**
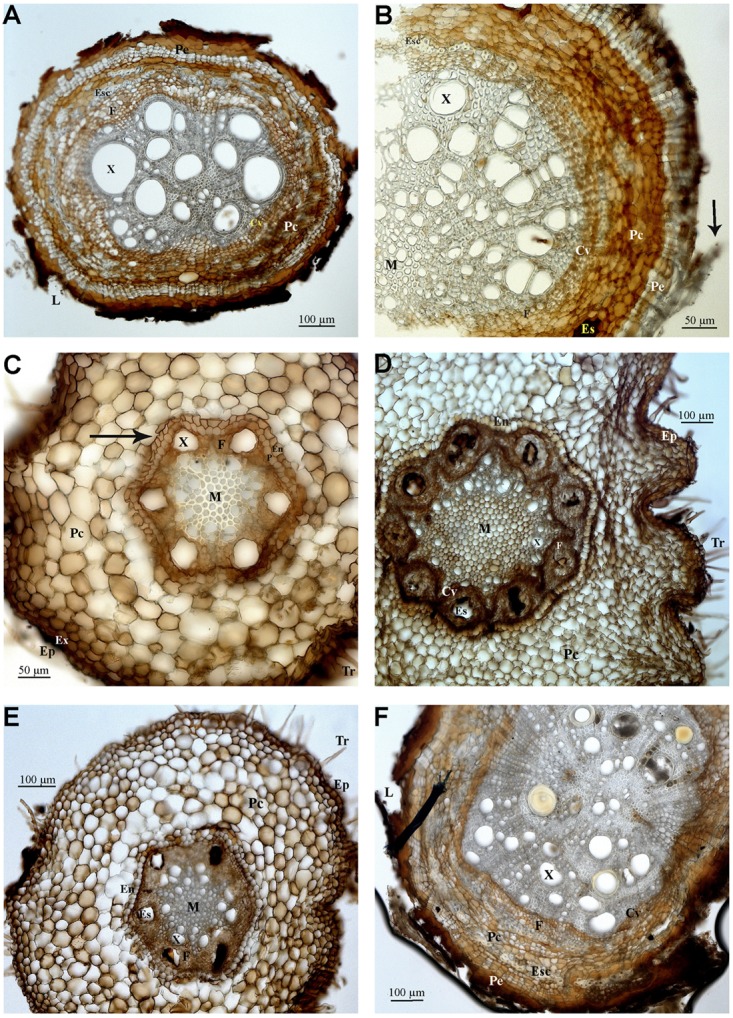
Photomicrographs of transverse sections of Anacardiaceae **(A–E)**, and Sapindaceae **(F)** roots. **(A)** Sample 8, secondary growth. **(B)** Sample 9, secondary growth. **(C)** Sample 10, primary growth. **(D)** Sample 11, primary growth. **(E)** Sample 13, primary growth. **(F)** Sample 14, secondary growth. Abbreviations: Tr, root hair; Ep, epiderm; Ex, exoderm; Pe, periderm; Pc, cortical parenchyma; Cv, vascular cylinder; En, endoderm; P, pericycle; M, medulla; X, xylem; F, phloem; Es, secretory structure; Esc, schlerenchyma; L, lenticel. Arrows indicate B, sheathing suber; C, Caspary striations.

Cylindric root displaying primary growth (Samples 10, 11, 13 – **Figures 4C–E**): epiderm with root hairs, papillous cells, and cuticle deposition (**Figures 4D,E**), cortex with several layers limited internally by the pericycle, endoderm, and vascular cylinder. The peripheral area of the cortex is formed by parenchymal cells that increase in diameter closer to the vascular cylinder. The endoderm cells are quadrangular and compact, sometimes with U (**Figures 4C,E**) and O (**Figure 4C**) shaped Caspary striations. The pericycle (**Figures 4C,E**) has a layer of thin walled cells. The sectioned area shows vascular cambium cells, forming an arrangement where the phloem and possible secretory structures can be observed (**Figures 4D,E**). The root is hexarch (**Figures 4C,E**), the xylem is circular/irregular with wide lumen, with some cells in strands spread toward the fibrous pith, with polygonal cells (**Figure 4D**).

Elliptic or cylindric secondary growth roots (Samples 8, 9 – **Figures 4A,B**): periderm with scaling suber or with lenticels and central cylinder with xylem, rays, and phloem. The cortex displays several layers internally limited by the pericycle, endoderm, and vascular cylinder. Cortex displays a sheath or sclerenchymal ring with ramiform punctuations and parenchymal cells intermixed by islands of sclerenchymal fibers and secretory structures are present. Vascular cambium has irregular thin walled cells in comparison to the pith and sclerenchymal fibers. Xylem has main circular cells with wide lumen and fibrous pith.

### Sample 12 – (Apocynaceae subf. Apocynoideae)

Cylindric root displaying primary growth (**Figure 3C**): epiderm, cortex with several layers limited internally by the pericycle, endoderm, and vascular cylinder. The peripheral area of the cortex is formed by parenchymal cells that increase in diameter closer to the vascular cylinder. The endoderm cells are hexagonal. The pericycle has a layer of thin walled cells. Phloem is protected by fibrous shields with cellulose, lignine or suberine deposits.

### Sample 14 – *Serjania* (Sapindaceae)

Oblong root displaying secondary growth (**Figure 4F**): periderm with scaly suber and central cylinder with xylem, rays ad phloem. The cortex has many layers that are limited internally by the pericycle, endoderm, and vascular cylinder. A sclerenchymal ring with ramiform intercalar punctuations and parenchymal cells surrounding islands of sclerenchymal fibers and secretory structures is visible in the cortex. The vascular cambium is formed by three to five layers of rectangular cells. The xylem includes circular cells and medulla fibers.

### Sample 16 – *Vismia gracilis* (Hypericaceae)

Cylindric root displaying secondary growth (**Figure 3D**): periderm with lenticels and central cylinder with xylem, rays, and phloem. The cortex displays a sheath or sclerenchymal ring with ramiform punctuations, parenchymal cells intercalated with sclerenchymal fibers. The xylem cells are rounded.

## Discussion

### Barcoding Effectiveness to Aid Plant Fragment Identification

The identification of sterile or incomplete plant fragments represents a challenge for taxonomists who traditionally rely on reproductive structures and complete material to identify plant families, genera, and species. Genetic information databases, such as GenBank, also rely on the work of taxonomists as they are based on authenticated vouchers that are constantly studied and updated and made available from biological collections to the global scientific community (Suarez and Tsutsui, 2004).

Considered of great utility to identify both animal (Wells et al., 2001) and plant fragments (Howarth et al., 2007), DNA barcoding has been effective to identify sterile or incomplete specimens found within caves. Amongst the markers used here, three of the most commonly used ones for plants, we found that ITS2 sequences were more reliable, as they show higher specificity and can separate groups at lower taxonomic levels. Meanwhile, *rbc*L is a good marker for family and genus level, its sequence evolution rate is low and it does not discriminate between species (Li et al., 2015).

Furthermore, the majority of ITS2 sequences were amplified apart from two samples, while we did not have such good results with the *rbc*L and *trn*H-psbA regions. Therefore ITS2 can be considered a reliable marker to provide plant fragment identification (Howarth et al., 2007), and in general we found higher identity and query coverage values for ITS2 matches.

### Considerations on the Plant Species Retrieved and Their Habitat

**FIGURE 5:**
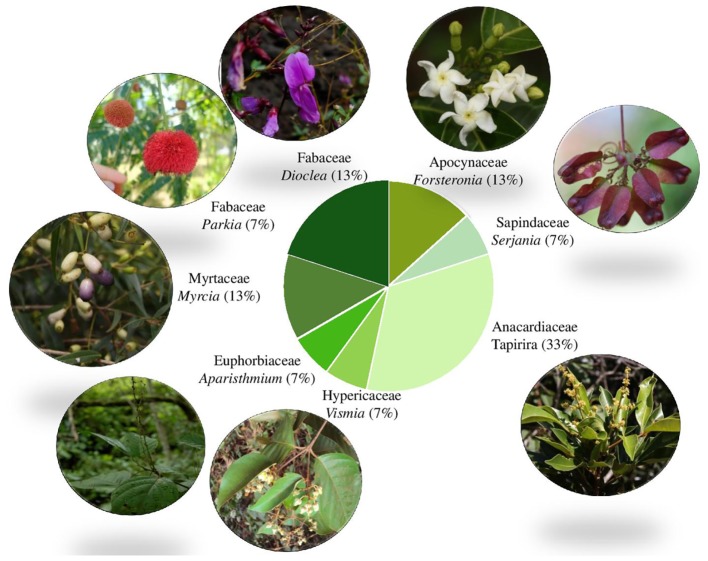
Proportion of samples per family showing images of the retrieved genera based on ITS2 DNA barcoding. Image credits (clockwise from top): *Forsteronia affinis* (N.F.O. Mota), *Serjania* (J.M. Rosa), *Tapirira guianensis* (W. Milliken), *Vismia gracilis* (L. Marinho), *Aparisthmium cordatum* (W. Milliken), *Myrcia sylvatica* (W. Milliken), *Parkia platycephala* (D. Zappi), *Dioclea apurensis* (D. Zappi).

Rhizothemes are woody structures and thus are more likely to belong to trees, large shrubs or woody vines or lianas. With the exception of two samples belonging to the Asterid clade, order Gentianales (Apocynaceae), all barcodes identified with ITS2, *rbc*L, and *trn*H-*psb*A belong to eudicots of the Rosidae clade, within four different orders: Malpighiales, Fabales, Myrtales, and Sapindales (The Angiosperm Phylogeny Group [APG], 2003). This may reflect the fact that Rosidae include more woody taxa than the Asterids (Smith and Donoghue, 2008). Plant families and genera retrieved can be seen in **Figure 5**.

*Myrcia* specimens found were, respectively, a fruit and a seedling. All samplings collected near the entrance of the cave belonged to genus *Vismia*. It is likely that the juicy fruits of both species were carried into the cave by bats or birds. The cave where these fragments were collected is home to a colony of frugivorous *Carollia perspicillata* bats (Prous *pers. comm*.).

The only complete, but vegetative collection was pressed (*Giulietti* 2576) and matched with the genus *Vismia* (Hypericaceae). The specimen was then shown to a specialist in the group who identified it as *Vismia gracilis* (Marinho, 2017).

Several root samples were retrieved as small trees belonging to *Tapirira*. Amongst the species of this genus, one was indicated by the barcode match (ITV3880) as belonging to *Tapirira guianensis*, a widespread woody species recently treated in the local flora by Hall and Gil (2017).

Once the putative identity of the roots is ascertained, it becomes possible to envisage the growth habit of the aerial part of these specimens. It was found that the roots projected deep into the caves do not belong exclusively to large forest trees as one may have expected (*Parkia*, that has both large tree and shrubby to treelet representatives) but to several treelets (*Tapirira*, *Vismia*), and also to woody lianas (such as either *Forsteronia*/*Secondatia/Odontadenia*; *Dioclea*; *Serjania*) that are common in the local forest that is classified as ombrophilous open forest with lianas and locally known as “mata-de-cipó.” These same taxa are also known to occur in woody vegetation assemblages or copses (*capões*) that grow where erosion causes soil formation and accumulation on the *canga* surface.

Unlike what was seen in the root material observed (**Figures 2D,E**), where root-caps are very obvious, the rhizothemes studied lacked root-cap and extension area between the tip of the structure and the piliferous region. However, the micromorphology of the following rhizotheme samples (Samples 4, 6, 8–14, and 16) has confirmed that all studied structures are roots, either in primary (**Figures 3A–C, 4C–E**) or secondary growth (**Figures 3A,D, 4A,B,F)**.

The presence of dark patches seen in all samples may be related to the presence of phenolic compounds, known protection against herbivory (Schaller, 2008). Secretory cells were spotted in Sample 4 (*Dioclea*/*Abrus* – Fabaceae), and *Tapirira* (Anacardiaceae), however, it was not possible to identify the type of substance or the type of secretory structure (duct or channel). Commonly known as “fruto de pombo” and belonging to the same plant family as cashew and mango, species of *Tapirira* produce a turpentine scented resin. According to Metcalfe and Chalk (1950), the Anacardiaceae are anatomically homogeneous, with cortex showing continuous or interrupted sclerenchyma and channels with wide secretory structures and phloem fibers, in coincidence with samples identified as Anacardiaceae through DNA barcoding.

Root nodules characteristic of the Fabaceae were not observed in the samples by micromorphology studies. It is known that under abundance of nitrites and nitrates, nodulation may be reduced or suppressed (Sprent, 2009), and that the availability of nitrogen compounds in caves is indicated as high, due to the presence of decomposer organisms and of guano deposited by bats (Ruchkys et al., 2015). Lenticels were observed in Sample 4 (*Dioclea/Camptosema/Abrus –* Fabaceae) and 8 (*Tapirira*, Anacardiaceae), 14 (*Serjania* – Sapindaceae) e 16 (*Vismia* – Hypericaceae) related to root respiratory needs (Fahn, 1979). Tannin and lenticels on the bark and oily substances are listed by Metcalfe and Chalk (1950) for young Fabaceae roots, linked with the brown color of undyed specimens.

Sample 14 (**Figure 4F**), identified as Sapindaceae (*Serjania*) matches closely the micromorphologic characters of this family, namely the general disposition of the cells, with distinct parenchyma and sclerenchyma cells and well defined vascular cambium and many cortical layers (**Figure 3A**) (Metcalfe and Chalk, 1950; The Angiosperm Phylogeny Group [APG], 2003).

## Conclusion

While working with lava tubes, Howarth et al. (2007) identified roots found in cavities and found their presence and abundance were important to maintain cave animal life including troglobites (arthropodes in that case), and had implications in cave conservation. They sequenced plants found above and inside the caves and matched their identity. This was feasible because their study area in Hawaii was a much more homogenous forest, involving only seven woody species of which three were native to the region. The present study was carried out in an environment of tropical biodiversity where there can be dozens of woody species growing above and in the surroundings of each one of the caves, and where the floristic composition also varies between sites. The inventory effort required to survey and identify the trees growing on and around each cave one by one would take many years, but the pre-existence of a DNA bank dealing with regional plants was fundamental in order to narrow the possibilities and provide insights on the identity of the roots found during this study. Despite the small number of caves sampled, there appears to be some specificity linked to the study sites, for instance the roots found in the one of the Serra da Bocaina caves were predominantly of *Tapirira* (Anacardiaceae) while the Serra Norte caves have at least two species of Fabaceae. The micromorphological approach, meanwhile, was useful to determine the nature of the rhizothemes (root or stem), concluding that all specimens collected during this work were roots, and also to support the DNA barcoding when conflicting identifications were retrieved. The local DNA barcode library was constructed as part of an effort to study the *canga* environment (Viana et al., 2016). Expanding this effort to forest species will improve the capacity to identify the species of plants that are part of the ferruginous cave environment.

Roots are believed to be a highly relevant resource for fauna inhabiting iron-ore cave ecosystems (Ferreira, 2005; Jaffé et al., 2016). The identification of the species that root inside the cave ecosystem is fundamental to foresee possible effects of anthropic interference on the surrounding environment and how it may affect the trophic resource contribution for the caves. It is expected that different plants species have specific responses to the environmental changes. Thus, species identification through DNA barcoding allows identifying which species actively participate in cave ecosystem, making it possible to manage the above ground environment. The knowledge of the associated taxa will contribute design management and conservation actions that help minimize negative impacts and safeguard subterranean biodiversity.

## Author Contributions

AR co-designed and initiated this study, prepared the micromorphology slides, extracted and analyzed the barcode data with support from GO, GN, and SV, and contributed to integration and interpretation of the data and final manuscript. DZ co-designed and continued this study, contributed to integration and interpretation of the data, prepared the first version of the manuscript, and contributed to the final manuscript. GN conducted and documented the bulk of the barcode analyses, integration and interpretation of the data, and contributed to the final manuscript. MW contributed to the barcode analysis, integration and interpretation of the data, and final manuscript. SV contributed to integration and interpretation of the data and final manuscript. MD contributed to the DNA extraction integration and interpretation of the data. RJ contributed to integration and interpretation of the data and final manuscript. XP contributed to the final manuscript, adding expertise on caves to the discussion. TG contributed to the organization and flow of the final manuscript. GO co-designed this study, contributed to the barcode analysis, integration and interpretation of the data, and final manuscript. AG co-designed this study, and contributed to the final manuscript.

## Conflict of Interest Statement

The authors declare that the research was conducted in the absence of any commercial or financial relationships that could be construed as a potential conflict of interest.

## Supplementary Material

TABLE S1 Complete sample Barcoding with identity values.

TABLE S2 Equivalent BOLD and GenBank codes for the samples and plant parts extracted.
